# Interdomain contacts control folding of transcription factor RfaH

**DOI:** 10.1093/nar/gkt779

**Published:** 2013-08-29

**Authors:** Sushil Kumar Tomar, Stefan H. Knauer, Monali NandyMazumdar, Paul Rösch, Irina Artsimovitch

**Affiliations:** ^1^Department of Microbiology, The Ohio State University, Columbus, OH 43210, USA, ^2^The Center for RNA Biology, The Ohio State University, Columbus, OH 43210, USA and ^3^Lehrstuhl Biopolymere und Forschungszentrum für Bio-Makromoleküle, Universität Bayreuth, Universitätsstraße 30, 95447 Bayreuth, Germany

## Abstract

*Escherichia coli* RfaH activates gene expression by tethering the elongating RNA polymerase to the ribosome. This bridging action requires a complete refolding of the RfaH C-terminal domain (CTD) from an α-helical hairpin, which binds to the N-terminal domain (NTD) in the free protein, to a β-barrel, which interacts with the ribosomal protein S10 following RfaH recruitment to its target operons. The CTD forms a β-barrel when expressed alone or proteolytically separated from the NTD, indicating that the α-helical state is trapped by the NTD, perhaps co-translationally. Alternatively, the interdomain contacts may be sufficient to drive the formation of the α-helical form. Here, we use functional and NMR analyses to show that the denatured RfaH refolds into the native state and that RfaH in which the order of the domains is reversed is fully functional *in vitro* and *in vivo*. Our results indicate that all information necessary to determine its fold is encoded within RfaH itself, whereas accessory factors or sequential folding of NTD and CTD during translation are dispensable. These findings suggest that universally conserved RfaH homologs may change folds to accommodate diverse interaction partners and that context-dependent protein refolding may be widespread in nature.

## INTRODUCTION

RfaH is an operon-specific transcription factor that belongs to the family of NusG proteins, the only universally conserved transcription factors (1). RfaH is a virulence factor that activates expression of horizontally transferred operons in *Escherichia coli* and several other human pathogens. These operons encode conjugation functions, lipopolysaccharide core, O-antigen, capsules and cytolytic toxins such as hemolysin (2), suggesting that RfaH may play a role in virulence. Indeed, deletion of RfaH attenuates virulence in *Salmonella* (3), but RfaH effects on pathogenesis in other bacteria remain to be elucidated. RfaH function requires a specific DNA element called *ops* (operon polarity suppressor) in the transcribed leader regions of its target operons (4) and is mediated by simultaneous RfaH contacts with the transcribing RNA polymerase (RNAP) and the translating ribosome (5). RfaH inhibits the activity of Rho, which terminates transcription of horizontally transferred genes (6), by three mechanisms: (i) increasing RNAP processivity; (ii) excluding NusG, a general transcription factor that potentiates Rho-dependent termination, from the transcription elongation complex (TEC); and (iii) coupling transcription and translation, thereby blocking Rho access to mRNA (7). Together, these mechanisms bring about dramatic effects: RfaH stimulates expression of its target operons several hundred fold (7,8).

**Figure 1.**
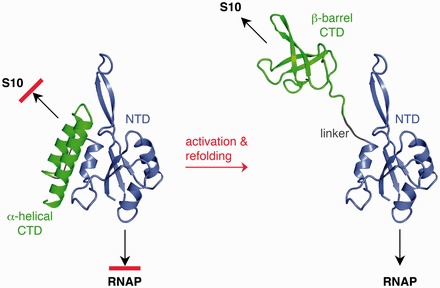
RfaH transformation. In the closed autoinhibited state, interaction surfaces for S10 and RNAP are masked. On dissociation, the RNAP-binding site on the NTD (green) becomes exposed, whereas the CTD (blue) refolds from an all-α into an all-β state that interacts with S10. The flexible linker is depicted as a gray line. Arrows indicate interaction partners of each domain. PDB IDs: 2OUG, RfaH; 2LCL, CTD. The figure was created using PyMOL (9).

RfaH antitermination function depends on a dramatic structural transformation (4). RfaH consists of two domains (Figure 1), the N-terminal domain (NTD) and the C-terminal domain (CTD). While the RfaH NTD shows the structural features typical for the NTD of NusG proteins, the CTD is an α-helical hairpin, in contrast to all other known NusG proteins where the CTD forms a five-stranded β-barrel. Additionally, RfaH CTD and NTD form a tight interface in the free protein. This is also untypical for members of the NusG family, as the two domains normally show no interaction (1,10); the only other known example where CTD and NTD interact is NusG from *Thermotoga maritima;* however, in this case, the CTD is in β-barrel conformation (11), rendering the closed state of RfaH unique.

In this closed form, the α-helical CTD (CTD-α) masks an RNAP-binding site on the NTD (12). During RfaH recruitment, transient interactions between the NTD and the TEC paused at the *ops* site are thought to trigger domain dissociation, allowing the NTD to establish contacts with RNAP that persist throughout transcription (13). After losing its contacts with the NTD, the CTD spontaneously and completely refolds into an SH3 β-barrel (CTD-β), which is essentially identical to the NusG CTD (4). While the NTD is responsible for the universally conserved antitermination modification of RNAP by RfaH (1) and its homologs, it is the CTD that confers the unique properties of RfaH. Each of the alternative folds of the CTD is essential for the proper control of gene expression. In the α-helical state, the CTD restricts RfaH action to *ops*-containing operons, avoiding interference with its paralog, the ubiquitous NusG (12,13). In the β-barrel state, the CTD interacts with the ribosomal protein S10 to enable translation in the absence of canonical ribosome recruitment elements (4). The two CTD states possess completely distinct well-defined folds and secondary structure elements, each conferring a characteristic regulatory property, classifying RfaH as the first member of the class of transformer proteins (14).

The β-barrel appears to be a preferred state of the CTD. The CTD folds as a β-barrel in the structures of all other known NusG homologs (1) and when expressed separately or when released from the RfaH NTD on proteolytic cleavage of the interdomain linker (3). This raises the question of how the CTD is induced to fold into the α-helical state during translation of the full-length RfaH. One possibility is that the CTD-α forms only when assisted by transient interactions between the NTD and the emerging CTD, potentially facilitated by chaperones or by ribosome pausing within the C-terminal region of *rfaH* mRNA. Examples of ribosome pausing determining protein folding pathways have been reported (15) and a trigger-factor dependent pause was detected by ribosome profiling in *rfaH* (16). Alternatively, the CTD may initially fold as a β-barrel and subsequently become converted into the α-helical form in the presence of the NTD. This idea is supported by equal distribution of the two structural states of the CTD observed in an RfaH variant with an E48S substitution in the NTD designed to destabilize the domain interaction (5). Such an equilibrium has not been observed, however, in wild-type (WT) RfaH, suggesting that the β-barrel is at most poorly populated in the absence of the TEC.

In this work, we used two approaches to distinguish between these models. First, we tested whether denatured RfaH can spontaneously refold into the native closed state. Second, we tested whether an RfaH variant in which the order of the domains is reversed is functional. Our results indicate that all information necessary to control its fold is encoded within RfaH itself, whereas accessory factors or sequential folding of NTD and CTD during translation are not essential for folding of active RfaH.

## MATERIALS AND METHODS

### Production, purification and refolding of WT RfaH

*Escherichia coli* BL21 XJb (λDE3) cells harboring pIA238 (12) were grown in Lauria Broth (LB) supplemented with kanamycin at 37°C. Once the OD_600_ reached ∼0.6, cells were induced with 0.2 mM of *Isopropyl* β-D-1-thiogalactopyranoside (IPTG), grown for 4 more hours and harvested (centrifugation at 6000*g* 4°C for 15 min). For purification in denaturing condition, cells were suspended in lysis buffer [50 mM Tris–Cl (pH 7.8), 8 M Urea, 500 mM NaCl and 0.25 mM Dithiothreitol (DTT)] for 30 min at room temperature followed by mild sonication. The lysate was clarified by centrifugation for 30 min at 40 000*g*. The supernatant was loaded onto Ni-NTA (GE Healthcare, Piscataway, NJ, USA) resin preequilibrated in lysis buffer. Next, the column was washed with lysis buffer containing progressively decreasing amounts of urea. Finally, RfaH was eluted by a step gradient of 50–200 mM imidazole in 50 mM Tris–HCl (pH 7.8), 500 mM NaCl, 1 mM DTT. Eluted fractions were analyzed by sodium dodecyl sulphate-polyacrylamide gel electrophoresis, the fractions containing homogenous protein were pooled and dialyzed against storage buffer (10 mM Tris–HCl, 100 mM NaCl, 50% (v/v) glycerol, 0.1 mM DTT, 0.1 mM EDTA) and stored at −80 or −20°C. Purification of native unlabeled WT RfaH, the isolated NTD and RNAP were carried out as described previously (12).

### Production and purification of domain-swapped RfaH

Plasmid pIA1181 encodes for His6-MP_112_-L_162_PKDIVDPATPYPGQ_2_-K_100_ domain-swapped (DS) RfaH. Here, subscript numbers denote the positions of respective amino acids in the WT RfaH. *Escherichia coli* BL21 XJb (λDE3) cells harboring pIA1181 (encoding His_6_-tagged DS RfaH) were grown at 37°C in LB containing kanamycin till OD_600_ reached ∼0.6, induced with 0.2 mM IPTG and grown for 14–16 h at 18°C. Cells were harvested by centrifugation at 6000*g* 4°C for 15 min and lysed in 50 mM Tris–HCl (pH 7.9), 50 mM NaCl, 5% (v/v) glycerol, 1 mM β-mercaptoethanol and EDTA-free protease inhibitor (Roche, Indianapolis, IN, USA). Lysate was clarified by centrifugation for 30 min at 40 000*g* and protein was purified on nickel affinity and Superdex 75 columns (GE Healthcare). ^15^N-labeled WT RfaH and the isolated CTD were purified as described previously (5); for ^15^N-labelled DS RfaH, the procedure of WT RfaH was used.

### NMR spectroscopy

Spectra were recorded on Bruker Avance 700 MHz or Avance 800 MHz spectrometers with cryogenically cooled triple-resonance probes equipped with pulsed field-gradient capabilities. NMR data were processed using in-house routines and visualized using NMRView (17). [^1^H,^15^N]-heteronuclear single quantum correlation (HSQC) spectra of ^15^N-WT RfaH and ^15^N-DS RfaH were recorded at 288 K, spectra of ^15^N-RfaH CTD were recorded at 298 K. ^15^N-WT RfaH and ^15^N-DS RfaH were in 10 mM potassium phosphate buffer (pH 7.5), 50 mM KCl. All samples contained 10% (v/v) D_2_O.

In the refolding experiments of ^15^N-RfaH and ^15^N-RfaH-CTD [^1^H,^15^N]-HSQC spectra in refolding buffer [25 mM Tris–HCl (pH 7.5), 150 mM NaCl] were recorded before denaturation. Urea was then added to 8 M over a period of 30 min on ice, followed by an incubation of further 30 min on ice. Five hundred microliters were then centrifuged for 10 min at 16 000*g* at 4°C to remove precipitate before the sample was analyzed by NMR spectroscopy. Proteins were refolded by stepwise dialysis at room temperature against refolding buffer containing 4, 2, 1 M urea and no urea; each dialysis step took at least 2 h. The final dialysis step was carried out overnight and the samples were then concentrated using VivaSpin devices (Molecular Weight Cut Off (MWCO) 3 kDa; EMD Millipore, Billerica, MA, USA). The refolded protein samples were centrifuged for 10 min at 16 000*g* and 4°C before analyses by NMR spectroscopy.

### Pause assays

Thirty nanomolar linear DNA template generated by polymerase chain reaction amplification, 40 nM holo RNAP, 100 µM ApU, starting NTP subsets (5 µM each of ATP and GTP, 1 µM CTP and 10 µCi [α^32^P]-CTP, 3000 Ci/mmol) were mixed on ice in TGA10 buffer (20 mM Tris–acetate, pH 7.9, 20 mM Na acetate, 10 mM MgCl, 5% glycerol, 0.1 mM EDTA and 2 mM β-mercaptoethanol). Halted radiolabeled TECs were formed by incubation at 37°C for 15 min. These were further mixed with 50 nM RfaH variants for 3 min. Elongation was started in presence of 25 µg/ml rifapentin by the addition of NTPs mix containing 150 µM of each ATP, CTP and UTP and 10 µM GTP. Aliquots were withdrawn at 7, 15, 30, 60, 120, 240, 480, 960 and 1200 s. Reactions were stopped by the addition of an equal volume of STOP buffer (10 M urea, 50 mM EDTA, 45 mM Tris–borate, pH 8.3, 0.1% bromophenol blue and 0.1% xylene cyanol). Samples were heated for 2 min at 90°C and were separated on 6% acrylamide (19:1), 7 M urea, 0.5× Tris-borate-EDTA (TBE) gels. The gels were dried, and RNA products were visualized by Typhoon FLA 9000 scanner and quantified using Image Quant software (GE Healthcare).

### Reporter assays

The *lux* reporter vector pIA1087 (4) was co-transformed with the plasmids encoding His_6_-tagged WT (pIA957) or DS (pIA1183) RfaH under the control of the P_BAD_ promoter, or an empty vector into a Δ*rfaH* strain (18) and plated on media containing 10 µg/ml tetracycline and 100 µg/ml carbenicillin. Single colonies were inoculated into 2 ml LB supplemented with antibiotics and incubated with agitation overnight at 37°C. The cultures were diluted into fresh LB with antibiotics to an OD_600_ of ∼0.05 and grown at 37°C for additional 6 h. Background expression from P_BAD_ was sufficient to generate readily measurable signals. Luminescence was measured in 200 µl aliquots in triplicates on FLUOstar OPTIMA plate reader (BMG Labtech, Ortenberg/Germany) and normalized by cell density.

### Western blotting

Cells were grown under the same conditions as for the *lux* reporter assay, collected by centrifugation, resuspended in 700 µl of 50 mM Tris–HCl, pH 7.9, 100 mM NaCl, 5% glycerol, 0.1 mM DTT, 0.5 mg/ml lysozyme and disrupted by sonication. The extracts were cleared by centrifugation, and total protein concentrations were determined by Bradford assay. Sample lanes contained ∼30 µg protein; for control lanes, 40 ng of purified RfaH was used. These were separated on a 12% sodium dodecyl sulphate Bis–Tris gel (Invitrogen, Carlsbad, CA, USA). The proteins were transferred in Tris–glycine buffer, pH 8.3, containing 20% methanol onto Trans-Blot nitrocellulose membrane (Bio-Rad Laboratories, Hercules, CA, USA) at 18.2 V for 2 h in a semidry Genie blotter (Idea Scientific, Minneapolis, USA). The blots were blocked overnight in 1× PBS-T, pH 7.5, 0.2% Tween 20 containing 5% nonfat dry milk. Following incubation with rabbit polyclonal antibodies diluted 1:4000 in Phosphate-Buffered Saline-Tween (PBS-T) for 2 h at room temperature, the membrane was washed and probed with rabbit IgG (GE Healthcare) for 1 h (1:10 000 dilution in PBS-T), washed again and exposed to ECL detection reagents (GE Healthcare). Imaging was carried out on Chemi-doc XRS^+^ Molecular imager (Bio-Rad).

## RESULTS

### *In vitro* assay to probe the interdomain contacts in RfaH

**Figure 2.**
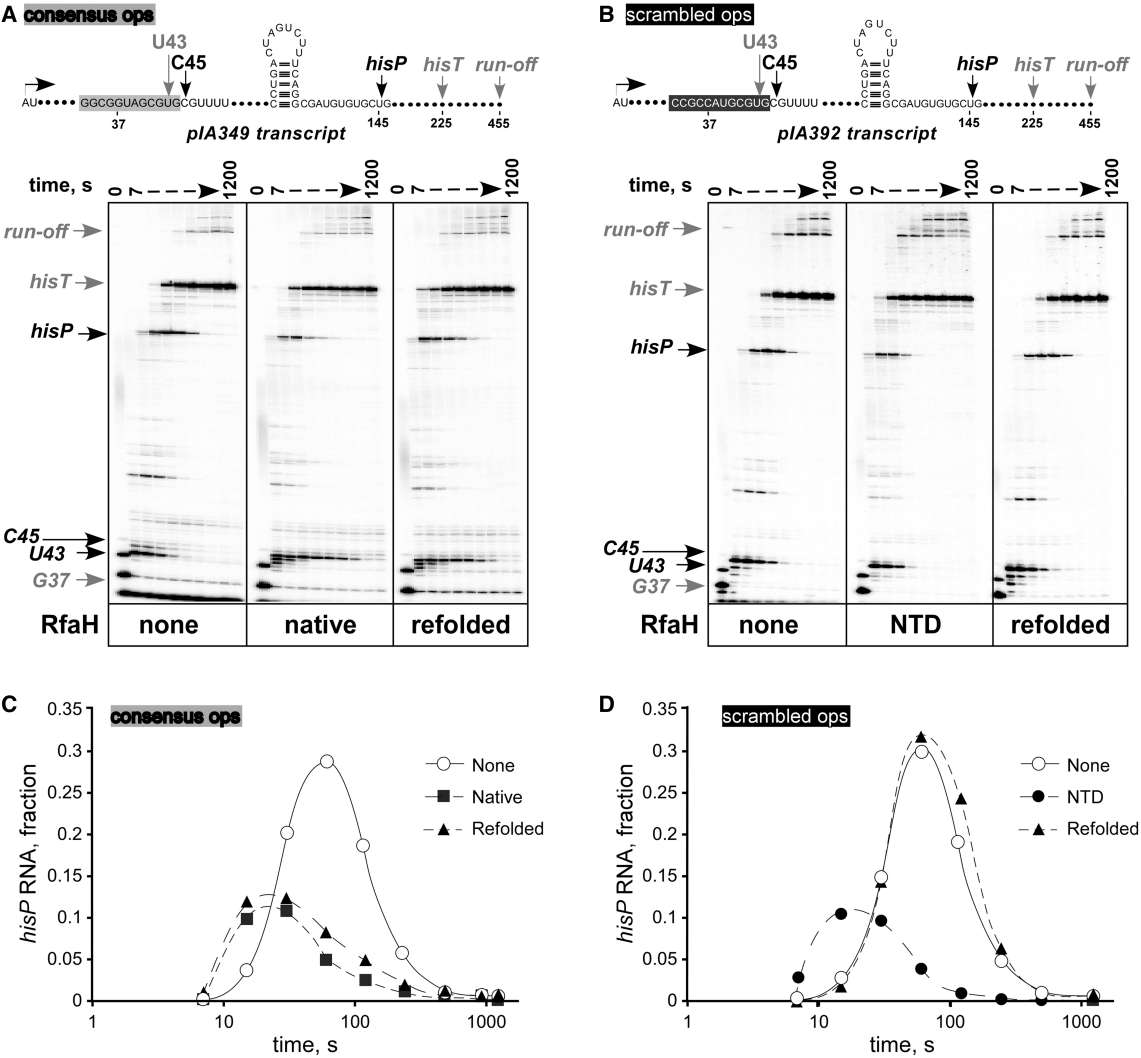
RfaH regains function after refolding. (**A**) Transcription pause assays on a linear pIA349 (4) template (shown on top) with the T7A1 promoter, the *ops* element, the start site (+1), transcript end (runoff), the pause sites that occur after the addition of U43, C45 and U145 (the *hisP* pause) and the *hisT* terminator indicated. Halted radiolabeled G37 TECs were preincubated with RfaH (native or refolded) at 50 nM or storage buffer for 3 min at 37°C, and then challenged with rifapentin at 25 µg/ml and NTPs (10 µM GTP, 150 µM ATP, CTP, UTP). Aliquots were withdrawn at times ranging from 7 to 1200 s and analyzed on a 6% denaturing gel. (**B**) Assays on a linear pIA392 template (4) lacking the *ops* site were carried out with the isolated NTD and refolded RfaH. (**C** and **D**) The fractions of RNA at the *hisP* from the gels in A and B, respectively, plotted as a function of time. The representative results from at least three independent experiments are shown; the absolute values vary within 10% between repeats, whereas the trends remain the same.

We first probed the folding state of RfaH using transcription templates that allow us to monitor the activity of the NTD and its interactions with the CTD in an *in vitro* system that uses only purified RNAP and RfaH (Figure 2). These templates encode a hairpin-dependent *his* pause (*hisP*) site positioned downstream of either an *ops* pause (*opsP*) element specifically recognized by RfaH (Figure 2A) or a scrambled *ops* site, which specifies an equally strong pause but does not interact with RfaH (Figure 2B). Once recruited to RNAP, the NTD reduces pausing at the *hisP* site. Recruitment of the closed-state RfaH in which the CTD-α masks the NTD requires the *ops* element to trigger domain dissociation; i.e. full-length RfaH is active only on *ops^+^* templates *in vitro* and *in vivo* (12,13). Conversely, if the CTD is absent (or is not properly folded and thus fails to mask the RNAP-binding site), the NTD can be recruited to the TEC at any site, and is thus able to reduce pausing on *ops*^−^ templates (12). Thus, comparison of RfaH antipausing activity on *ops*^+^ and *ops*^−^ templates provides an indirect assessment of the state of the CTD.

### RfaH spontaneously refolds into the active state

To test whether RfaH can correctly refold after denaturation, RfaH was absorbed onto Ni-NTA resin in the presence of 8 M urea, allowed to refold by gradually removing urea during the washing steps, eluted and dialyzed into a standard storage buffer (see ‘Materials and Methods’ section for details). Antipausing function of this ‘refolded’ protein was first tested on an *ops*^+^ template; RfaH purified under ‘native’ conditions (12) was used as a positive control. We found that both native and refolded proteins affected transcription similarly, delaying RNAP escape from the *opsP* site and reducing pausing at the *hisP* site (Figure 2). We note that the refolded protein was slightly less efficient in reducing pausing at the *hisP* site (Figure 2B and C), perhaps because it failed to completely refold. Because we used nearly equimolar concentrations of RNAP and RfaH (40 and 50 nM, respectively), even a small refolding defect would leave a fraction of RNAP unmodified.

To be able to support these functions, the compact NTD must establish three separate sets of contacts with three different parts of the TEC, namely with the nontemplate DNA strand, with the β gate loop and with the β′ clamp helices (β′CH). Thus, the refolded NTD appears to be folded properly. Next, we used an *ops*^−^ template to assess the state of the CTD. As described above, RfaH in the closed native state is not recruited to RNAP transcribing this template owing to failure of domain dissociation, and thus will fail to reduce pausing. We found that the isolated NTD, which was used as a control, reduced pausing on the *ops*^−^ template, whereas refolded RfaH (Figure 2B) or native RfaH (12) did not. Similarly, we showed that, in contrast to WT RfaH, the NTD activated expression of the *ops*^−^ *lux* reporter (18). These observations are consistent with the formation of the closed autoinhibited state of RfaH with CTD-α.

**Figure 3.**
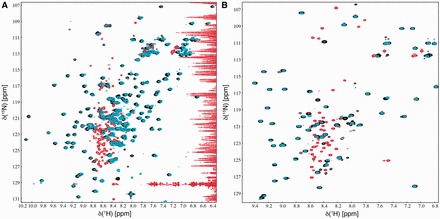
Denaturation and refolding of full-length WT RfaH (**A**) and RfaH-CTD (**B**). [^1^H,^15^N]-HSQC spectra of the ^15^N-labeled proteins before denaturation (black), after denaturation (red) and following refolding (cyan) are shown. The as-isolated proteins (172 µM WT RfaH, 239 µM CTD) were in 25 mM Tris–HCl (pH 7.5), 150 mM NaCl. To denature the proteins, urea was added to a final concentration of 8 M and [^1^H,^15^N]-HSQC spectra were recorded (18 µM WT RfaH, 101 µM CTD). Refolding was carried out by stepwise dialysis to remove urea and [^1^H,^15^N]-HSQC spectra of the refolded proteins were recorded (29 µM WT RfaH, 83 µM CTD). NMR experiments were conducted at 288 or 298 K for WT RfaH or CTD, respectively.

Together, our results suggest that RfaH spontaneously folds into the native state in which the NTD and CTD-α form a tight interface. However, transcriptional activity of RfaH serves only as an indirect reporter of the folding states of the NTD and CTD and does not allow one to ascertain that RfaH completely refolds into the native structure. Therefore, we visualized RfaH refolding directly using NMR spectroscopy (Figure 3). Initially, a [^1^H,^15^N]-HSQC spectrum of native ^15^N-labeled RfaH was recorded to ensure that the protein was correctly folded. Then RfaH was denatured in 8 M urea, which resulted in a [^1^H,^15^N]-HSQC spectrum typical of an unfolded protein, indicating that RfaH was completely denatured (Figure 3A). Subsequent stepwise removal of urea by dialysis finally yielded a [^1^H,^15^N]-HSQC spectrum nearly identical to the one of native RfaH with the CTD in the α-form. Neither before denaturation nor after refolding, an equilibrium of different conformations of the CTD could be observed. The refolding experiment was repeated with the isolated CTD, which is normally in the β-barrel conformation (Figure 3B). This protein domain could also be completely denatured in 8 M urea, and it returned to the β-state on urea removal as observed by NMR spectroscopy. This clearly shows that, on the one hand, both full-length RfaH and the isolated CTD fold spontaneously from a denatured state and, on the other hand, the CTD folds into the α-state only in the presence of the NTD.

In combination with the data of the *in vitro* transcription assays, these results indicate that RfaH regains its structure and functionality on removal of the denaturant and support the assumption that the presence of the NTD drives the CTD into the α-state and stabilizes it. However, they cannot exclude a possibility that co-translational folding may proceed at an accelerated rate.

### DS RfaH folds into a closed conformation

**Figure 4.**
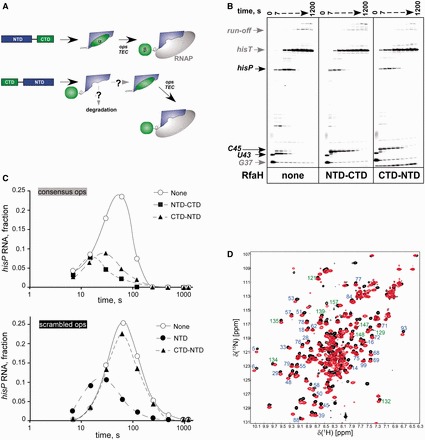
DS RfaH is active and folded. (**A**) Fates of the WT and DS RfaH. (**B**) Pause assays on pIA349, performed as in Figure 2A. (**C**) The fractions of *hisP* RNA from the panel A and Supplementary Figure S1, respectively, plotted as a function of time. The representative results from at least three independent experiments are shown; the absolute values vary within 10% between repeats, whereas the trends remain the same. (**D**) [^1^H,^15^N]-HSQC spectra of WT RfaH (black) and DS RfaH (red) were recorded at 288 K in 10 mM potassium phosphate buffer (pH 7.5) containing 50 mM KCl; both proteins were at 200 µM. Blue and green numbers correspond to the amino acids numbers of the NTD and CTD of WT RfaH, respectively.

An alternative approach to assess the requirements for co-translational folding of RfaH is to reverse the order of its domains in the amino acid sequence. Growing evidence suggests that although well-defined protein domains frequently fold as soon as they emerge from the ribosome exit tunnel, ribosome pausing at a key position may alter a folding pathway (15). For example, a completely folded NTD could interact with the N-terminal segment of the CTD and direct its folding into the α-helices. This templating action would be abolished if the order of domains were reversed (Figure 4A) because the β-barrel would likely fold before the NTD is made. In this case, one would expect that the CTD folds into the β-barrel when it emerges from the ribosome, just as it does when expressed alone. Subsequent translation and folding of the NTD would generate an open *ops*-independent form of RfaH (12,18) with a large nonpolar surface exposed. Given that the NTD cannot be overexpressed separately and precipitates when separated from CTD (at concentrations exceeding 2 µM), the open form is expected to be unstable in the cell. Only if the CTD can refold, the domain interface could be established, shielding the hydrophobic cavity on the NTD and conferring the requirement for the *ops* element.

We constructed an RfaH variant in which the order of domains is reversed, extending the linker by two residues relative to the WT RfaH to facilitate interdomain contacts. We found that this DS RfaH behaved similarly to the WT protein in *in vitro* transcription assays (Figure 4B and C); most importantly, it did not work on the template lacking *ops*, indicating that the RNAP-binding surface on the NTD is masked by the CTD that presumably is in α-helical form.

To ascertain that the DS RfaH assumes a closed state with the CTD in the α-form, we analyzed it by NMR spectroscopy. The [^1^H,^15^N]-HSQC spectra of the DS RfaH and WT RfaH were similar (Figure 4D), and signals typical for well-structured parts of the NTD and CTD-α of WT RfaH could be clearly found in the spectrum of DS RfaH. This allowed the assignment of residue numbers to signals from both the NTD and the CTD in the DS RfaH spectrum (e.g. residues 5, 6, 29, 33, 51, 53, 68, 69 84, 88 from the NTD and residues 121, 132, 134, 135, 147 and 157 from the CTD). Additional signals may be attributed to the additionally introduced amino acids and rearrangements in the linker region. These data indicate that DS RfaH is structurally similar to WT RfaH with the CTD in the α-helical state.

### DS RfaH is active *in vivo*

**Figure 5.**
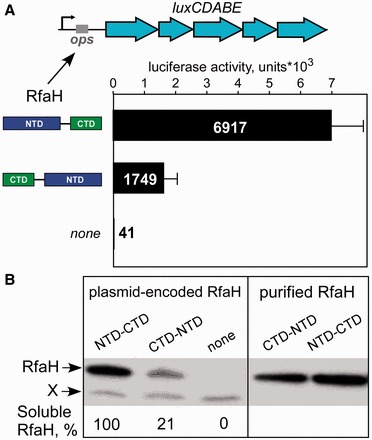
The DS RfaH is functional *in vivo*. (**A**) Expression of a reporter *lux* operon lacking a Shine-Dalgarno element activated by RfaH (WT or DS) encoded on a compatible plasmid. The results are an average ± standard deviation of three independent experiments. (**B**) Steady-state levels of RfaH measured by western blotting under the conditions identical to A. The band shown as X is an unknown protein that cross-reacts with polyclonal anti-RfaH antibodies.

Next, we tested whether DS RfaH is functional *in vivo*. We expressed at low levels the WT or DS RfaH variant in a Δ*rfaH E. coli* strain from a plasmid and measured the activity of luciferase encoded by the *luxCDABE* operon on a compatible plasmid. We previously showed that RfaH dramatically stimulates *lux* expression from vectors lacking a Shine-Dalgarno element (3), consistent with a key role of RfaH in coupling transcription to translation. The WT and the DS RfaH increased *lux* expression ∼170- and 50-fold, respectively, as compared with the empty vector (Figure 5A). The reduced (to ∼25%) DS RfaH activity could be due to defects in folding or to reduced steady-state protein levels, for example, if the ‘open’ RfaH is targeted for cellular degradation (Figure 4A). Consistent with the second scenario, Western blotting using polyclonal antibodies specific for the CTD (18) revealed that the DS RfaH was present at ∼20% level relative to the WT protein under conditions used for the reporter assays (Figure 5B). Our results demonstrate that the DS RfaH is fully active in the cell.

## DISCUSSION

RfaH CTD is able to transform from an α-helical hairpin into a β-barrel, and each state plays its specific regulatory role. Here, we asked whether RfaH CTD folding into the less stable α-helical state requires co-translational folding assisted by the ribosome or a ribosome-associate factor. We show that in the full-length RfaH, the CTD folds into an α-helical structure on refolding from the denatured state or even when synthesized by the ribosome before the NTD in the DS RfaH variant. In the latter case, we favor a scenario in which the CTD first folds into the stable β-barrel, just as it is known from the isolated CTD (5), and its transformation into the α-form is induced as soon as the NTD becomes available. We cannot exclude a possibility that in the DS RfaH, folding of the CTD is delayed until the completion of the entire chain; however, other SH3 domains fold on a time scale of 10 ms (19,20).These data demonstrate that co-translational folding is dispensable for the formation of the native RfaH fold and that the NTD, when tethered to the CTD, templates its folding into the α-helices.

**Figure 6.**
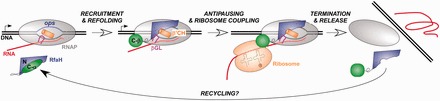
Refolding of the CTD could be reversible. Before recruitment, RfaH exists in a closed state, with the CTD-α masking the RNAP-binding site on the NTD. Following recruitment, RfaH opens up and the NTD establishes interactions with RNAP β gate loop (GL; magenta) and β′ clamp helices (CH; orange) elements, which mediate antitermination. The CTD is released and refolded, making contacts to S10 that are proposed to facilitate ribosome (or 30S) loading. Both sets of contacts are likely maintained throughout expression of the entire operon but then are sequentially broken when first ribosome, and then RNAP terminate synthesis. Once both domains lose contacts with their respective partners, the NTD can mold the CTD into the α-helix, thereby completing the cycle.

Our findings that the DS RfaH regains its native fold suggest that the closed form of RfaH could be regenerated after each cycle, when ribosome and RNAP dissociate from the nucleic acid chains at the end of an operon (Figure 6). If it remained bound to RNAP, RfaH would inhibit initiation because it competes with the σ factor for binding to the core enzyme (21). However, this scenario is unlikely because RfaH binds to the isolated RNAP weakly (4), and its persistent contacts with RNAP observed during elongation *in vivo* (13) are likely stabilized by a network of protein/DNA interactions. Likewise, RfaH contacts with S10 are weak in the absence of other components (4).

What happens to RfaH after dissociation? ChIP-chip data show that RfaH is restricted to *ops*-containing operons *in vivo* (13), indicating that the open form does not persist in the cell. Thus, RfaH is either rapidly degraded or refolded into the closed autoinhibited form. We favor the second scenario in which the CTD plasticity allows RfaH to be recycled after each use, as the results obtained with the DS RfaH suggest that the CTD can undergo a β→α switch. This conversion remains to be demonstrated but we note that RfaH is optimized for readily shifting structures—we showed that disruption of a single interdomain salt bridge was sufficient to achieve an approximately equal distribution between the two states in solution (4), implying that the α/β equilibrium may be shifted by ligands binding to either RfaH domain.

It is possible that other protein domains may have drastically different folds not predicted by homology modeling or even structures of individual domains if they are involved in interdomain interactions, interact with other proteins as part of a larger complex or bind an abundant ligand. In particular, our results suggest a tantalizing possibility that ubiquitous β-barrel domains, which are present in many proteins in addition to NusG-like factors, may undergo similar changes on binding to their partners. Our studies have broad implications for the understanding of structure, dynamics and evolution of proteins and underscore the limitations of studies of isolated protein domains.

## CONCLUSIONS AND PERSPECTIVES

The fold of a given protein is primarily dictated by its amino acid sequence, allowing for *de novo* prediction of protein structures (22). However, examples where short peptides (23) and small proteins (24) adopt different secondary and tertiary structures depending on a broader context have been characterized. Even more remarkably, some proteins, such as the chemokine lymphotactin (25) and Mad2 (26), can interconvert between two states with different topologies. In contrast to these Metamorphic proteins, which preserve some, if not most, elements of their secondary structures, the RfaH CTD undergoes a complete α→β switch to acquire a new role in translation (4). Similarly, the key molecular switch in prion diseases is a conversion of a monomeric α-helical cellular prion into an oligomeric infectious prion enriched in β-sheets, and one among three current models postulates a complete α→β prion conversion (27). Beyond contextual interactions with other segments of the same peptide chain, many extrinsic factors, such as ribosome pausing (15), protein chaperones (28) and even osmolytes (29), can modulate the folding pathway. In the crowded cellular environment, proteins fold while surrounded by many potential interactors, from small molecules to nucleic acids to other proteins, and scenarios where proteins adopt one of alternative folds on binding to a ligand appear to be common.

Together, these studies suggest that context-dependent changes of protein structure are widespread in nature and play key functional roles. In extreme cases, refolding could generate completely new interaction interfaces, which are masked in isolated structures. Serpins, for example, undergo large rearrangements on binding to their targets (30), and the RfaH CTD can only bind to S10 after total transformation from its α-helical state into a β-barrel (4). The ability to change its fold would greatly expand the regulatory repertoire of a given protein. We demonstrated that in the case of the transformer protein RfaH, all information as to which fold to adopt is encoded in the protein itself, rendering RfaH a paradigm for exploring emerging principles of protein plasticity.

## SUPPLEMENTARY DATA

Supplementary Data are available at NAR Online.

## FUNDING

Deutsche Forschungsgemeinschaft (DFG) [Ro617/18-1 to P.R.]; National Institutes of Health [GM67153 to I.A.]. Funding for open access charge: [GM67153 to I.A.].

*Conflict of interest statement*. None declared.

## Supplementary Material

Supplementary Data
