# Supplementary material for: Interdomain contacts control folding of transcription factor RfaH
Source: Nucleic Acids Res. 2013 Aug 29;41(22):10077–85. doi: 10.1093/nar/gkt779 (PMC3905879; doi:10.1093/nar/gkt779)
Supplement: Supplementary Data [file supp_41_22_10077__index.html]

Interdomain contacts control folding of transcription factor RfaH — Supplementary Data 

# Interdomain contacts control folding of transcription factor RfaH

## Supplementary Data

files

**Files in this Data Supplement:**

- Supplementary Data - pdf file
