# Supplementary material for: Interdomain contacts control folding of transcription factor RfaH
Source: Nucleic Acids Res. 2013 Aug 29;41(22):10077–85. doi: 10.1093/nar/gkt779 (PMC3905879; doi:10.1093/nar/gkt779)
Supplement: Supplementary Data [file supp_gkt779_nar-02012-v-2013-File008.pdf]

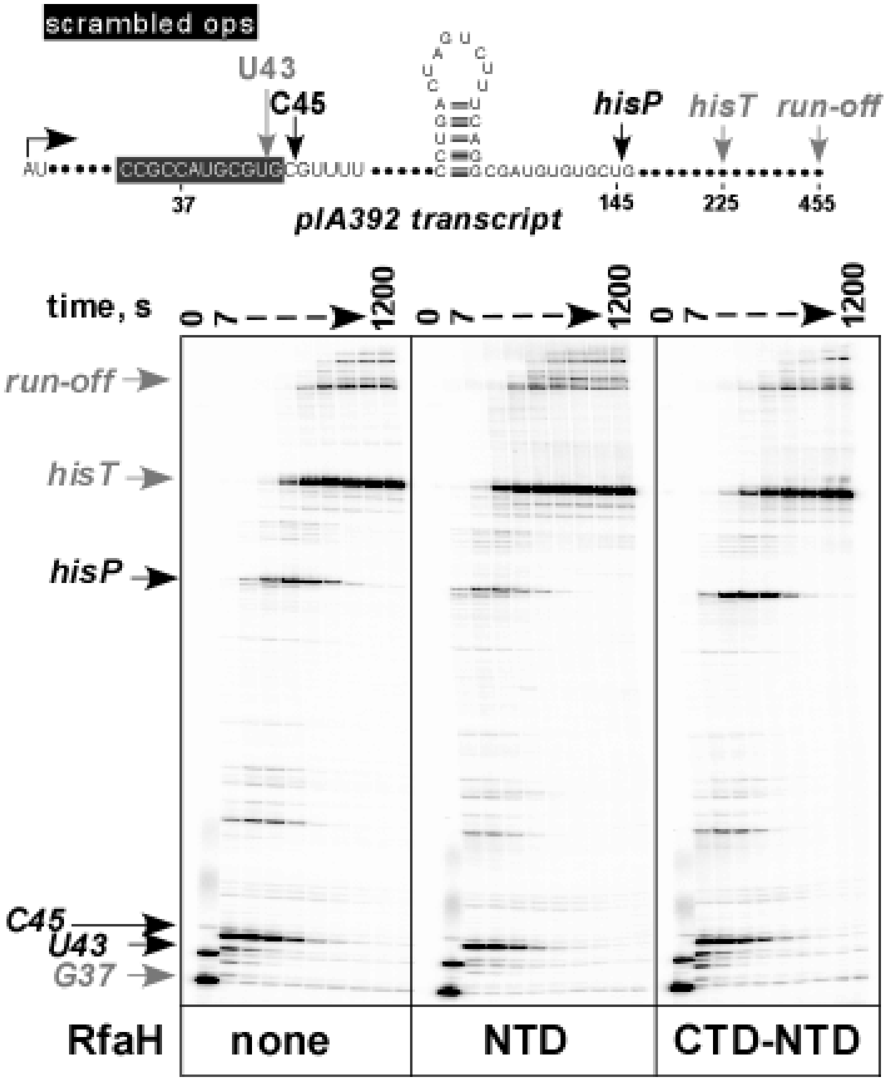

Supplementary Figure 1. Domain-swapped RfaH is *ops* specific. Transcription pause assays on a linear pIA392 (4) template (shown on top) with the T7A1 promoter, the *ops* element, the start site (+1), transcript end (run-off), the pause sites that occur after the addition U43, C45, and U145 (the *hisP* pause), and the *hisT* terminator indicated. Halted radiolabeled G37 TECs were pre-incubated with RfaH (DS or the isolated NTD) at 50 nM or storage buffer for 5 min at 37 oC, and then challenged with rifapentin at 100 µg/ml and NTPs (10 µM GTP, 150 µM ATP, CTP, UTP). Aliquots were withdrawn at times ranging from 10 to 960 sec and analyzed on a 6% denaturing gel. Quantification of these data is presented in Figure 4.
